# Chimp Optimization Algorithm Influenced Type-2 Intuitionistic Fuzzy C-Means Clustering-Based Breast Cancer Detection System

**DOI:** 10.3390/cancers15041131

**Published:** 2023-02-10

**Authors:** Prasanalakshmi Balaji, Vasanthi Muniasamy, Syeda Meraj Bilfaqih, Anandhavalli Muniasamy, Sridevi Tharanidharan, Devi Mani, Linda Elzubir Gasm Alsid

**Affiliations:** 1 College of Computer Science, King Khalid University, Abha 61421, Saudi Arabia; 2 Applied Science College, Mahala Campus, King Khalid University, Abha 61421, Saudi Arabia; 3 College of Science and Arts, Sarat Abidah Campus, King Khalid University, Abha 61421, Saudi Arabia

**Keywords:** cancer detection, clustering methods, magnetic resonance imaging, optimization, sensitivity, specificity

## Abstract

**Simple Summary:**

Breast Cancer Detection being an area of importance in detecting it in early stages and to assist the Oncologists in analyzing the stage and mode of further treatment, it gains equal importance to all types of cancer detection. Using the proposed optimization based machine learning model in detection process is supposed to help the patients and the oncologists in deciding further process.

**Abstract:**

In recent years, breast cancer detection is an important area of concentration towards curative image dispensation and exploration. Detection of a disease at an early stage is an important factor in taking it to the next level of treatment. Accuracy plays an important role in the detection of disease. COA-T2FCM (Chimp Optimization Algorithm Based Type-2 Intuitionistic Fuzzy C-Means Clustering) is constructed for detection of such malignancy with the highest accuracy in this paper. The proposed detection process is designed with the combination of type-2 intuitionistic fuzzy c-means clustering in addition to oppositional function. In the type-2 intuitionistic fuzzy c-means clustering, the efficient cluster center can be preferred using the chimp optimization algorithm. Initially, the objective function of the type-2 intuitionistic fuzzy c-means clustering is considered. The chimp optimization algorithm is utilized to optimize the cluster center and fuzzifier in the clustering method. The projected technique is implemented, and in addition, performance metrics such as specificity, sensitivity, accuracy, Jaccard Similarity Index (JSI), and Dice Similarity Coefficient (DSC) are assessed. The projected technique is compared with the conventional technique such as fuzzy c means clustering and k mean clustering methods. The resulting method was also compared with existing methods to ensure the accuracy in the proposed method. The proposed algorithm is tested for its effectiveness on the mammogram images of the three different datasets collected from the Mini–Mammographic Image Analysis Society (Mini–MIAS), the Digital Database for Screening Mammography (DDSM), and Inbreast. The accuracy and Jaccard index score are generally used to measure the similarity between the proposed output and the actual cancer affected regions from the image considered. On an average the proposed method achieved an accuracy of 97.29% and JSI of 95%

## 1. Introduction

Breast cancer is the world’s most deadly disease affecting women. It starts with unregulated cell division, which leads to the growth of a tumor in the breast [1]. Anomalies, changes in the skin tone of the chest, and the change in shape of a breast at extreme cases are some symptoms of breast cancer or malignant growths of the chest that are noticeable. Imaging modalities such as ultrasound rays and appealing visuals, in general, decompress the specific details of the breast image. However, mammography, which employs the lower region of the X-rays for image creation, is the greatest technique to identify breast cancer in the early stages. Mammography can discriminate abnormalities like calcification and lumps, as well as other ambiguous signs including engineering degeneration and bilateral imbalance [2]. At the mammogram address, a mass, thickness, lump, or deformity is predicted. Smoothness and irregularity, for example, are often complementary to mammogram images. Breast cancer images are analyzed using a variety of imaging techniques, including CT scans, PET, spectroscopy, SPECT, and MRI [3].

Image detection in breast cancer is utilized to organize the disease or is mass pixels that can be used to identify a mammogram, careful orchestration, and image-aided suggestions. In order to filter out suspicious breast images from a whole sum of data collected, artificial intelligence model developed was used [4]. In another fuzzy-level set calculation aiming at computerized breast cancer image identification, the Fuzzy ensemble serves as the basic level synthesizer. The spatial data have approached a move in this manner, and limited leakage can be avoided. Breast cancer image division is a common difficulty in the new projected mammogram division technique focused on MRI images. To organize each voxel into distinct classes, the approach employs an adjacent autonomous project-based sequencing strategy [5].

In a fuzzy C-means clustering system, the MRI image detection approach to K-means clusters the exhibition. To give proper cerebral breast cancer disease detection, thresholding and level set partition locations are followed. For the picture detection of the K-means group, this method can obtain sections of unbounded computing time. Similar to ambiguous C-references in precision pieces, it can attain the best conditions. The adaptive controlled strip-based MRI image cerebral mammogram segment obscure C-marker boxing system demonstrated potential improvements for local system adaptation [6]. It limits image sophistication, as well as ability to set collection boundaries and lower computing expenses. These approaches, however, do not produce the best-fragmented effects on MRI images [7]. The clustering approach has recently been combined with Artificial Intelligence (AI) techniques such as Grey Wolf Optimization, Particle Swarm Optimization [8], and Whale Optimization algorithms [9] to obtain the best-detected outcomes in MRI images.

To monitor complex queries in methodology and procedures, AI computations have received a lot of attraction and responded with suitable and accurate responses. AI-based techniques have shown remarkable performance in a variety of image recognition problems [10]. The employment of different component extraction technologies, mammography datasets, and AI modeling has shown the difference in the research techniques adopted.

## 2. Related Works

Numerous such techniques are adopted to work on breast cancer images towards detection of malignant status in order to facilitate further diagnostic and treatment procedures. A framework is created [11] based on the transfer learning principle. In addition, a variety of augmentation procedures, including multiple rotation combinations, scaling, and shifting, were consumed to prevent overfitting and achieve consistent outcomes by increasing the number of mammographic images. The suggested system was tested on the dataset of the Mammographic Image Analysis Society (MIAS), and it obtained an accuracy of 89.5 percent using ResNet50 and 70 percent using the Nasnet-Mobile network. Pre-trained classification networks were shown to be much more successful and efficient in the proposed method, producing them additional satisfaction in favor of medicinal imaging, especially for short preparation datasets. The transfer learning method is being used to distinguish between malignant and benign breast cancer by fine-tuning several pre-trained models.

Another transfer learning method developed [12] distinguishes between malignant and benign breast cancer by fine-tuning several pre-trained models to collect imaging data from 10 breast cancers in nine women who received a microwave imaging examination before surgery. The tumors ranged in size from 4 to 10 cm. Microwave imaging’s ability to identify breast cancer and characterize its position and size as compared to conventional mammography and pathological findings on a five-point scale by a radiologist specializing in breast imaging. To analyze a large number of patients and enhance cost-effectiveness, the data-collecting time must be considerably reduced. Furthermore, the reconstructed images should be reviewed immediately after the test, as body movement or equipment faults during the examination may hinder optimal image data gathering. The apparatus was found to be safe, and it looks like it may be used repeatedly. These findings indicate that the technology could be used to screen for breast cancer.

A breast cancer detection system is completely automated [13]. First, the U-Net network is used in the breast cancer detection model to automatically separate and isolate the breast area from the rest of the body, which acts as noise. Second, we propose a two-class deep learning model trained from scratch for categorizing normal and diseased breast tissues using thermal images. It is also utilized to extract more features from the dataset, which aids in the training of the network and improves the efficiency of the classification process. The three main parts of this method are resizing breast area segmentation and a deep learning model for classification. Thermal pictures are downsized to a reduced size to speed up computation during the resizing process. In the breast area segmentation step, a U-Net network is used to automatically recover the breast region. In the deep learning model for the classification phase, a deep learning model based on a two-class CNN that is built from scratch is employed for the categorization of normal and diseased breast tissue. The system’s feasibility is demonstrated by statistical analysis using the ANOVA test. Furthermore, since the suggested approach is domain-agnostic, it is capable of solving a wide range of computer vision issues.

Breast cancer sample classification using an MLP-based homogeneous ensemble technique is proposed [14]. The goal of ensemble learning is to improve the categorization process. To develop a new classifier, this method includes combining different fundamental classifiers. Several optimization approaches, including GA, PSO, and ODMA, have been tried in this field to see which algorithm gives the most optimal parameters for MLP. These parameters include effective features, the number of hidden layers, the number of nodes in layers, and weight values. The proposed algorithm is evaluated on three datasets from the Wisconsin Breast Cancer Database, and then it is compared against other algorithms to see which one is the most accurate. Optimization strategies for tweaking MLP neural network parameters are being developed. The creation of a homogeneous ensemble classification framework is based on the change of neural network parameters. Even more models were presented [15,16] and discussed related to the state of art. Some more models are used on the three datasets taken up for consideration, and their survey results are shown in the Table 1:

The method has been designed for the best detection of breast cancer disorders. Based on the studies conducted and considering the stages of implementation of all the previous methods, the workflow of the proposed work is given as follows.

- The proposed detection method incorporates type-2 intuitionistic fuzzy c-means clustering for clustering suspicious region from the mammogram. Initially, the type-2 intuitionistic fuzzy c-means clustering objective function is considered with the consideration of intuitionistic fuzzy data extracted from the MRI images.
- The chimp optimization approach can be used to identify the most efficient cluster center in type-2 intuitionistic fuzzy c-means clustering. Chimp optimization algorithm is utilized to optimize the cluster center and fuzzifier from the clustering method.
- The projected technique is executed in Python to evaluate the performance and similarity measure of the proposed system. The projected technique is compared with the conventional techniques such as fuzzy c means clustering and k mean clustering methods.

## 3. Proposed System Model

**Figure 1:**
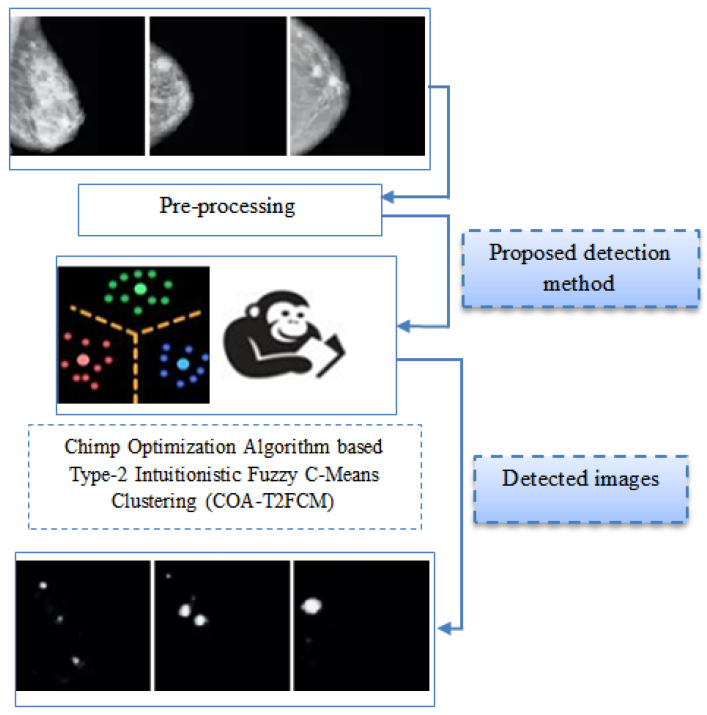
Proposed Breast Cancer Detection System.

Breast cancer detection from MRI images is a difficult operation due to irregularity in cohort of disease images in addition to its complex structure. Hence, in this paper, COA-T2FCM is developed to achieve optimal breast cancer detection. The proposed method contains two main phases: a pre-processing stage and the detection phase. In the primary phase, the pre-processing stage eliminates the unwanted noise present in the images, which is achieved by intensity normalization, orientation correction, and pectoral muscle removal. In the second phase, the detection phase detects the lump from the MRI images. Usually lumps in MRI images occur by means of diseases or abnormalities. The proposed method’s entire structural design is depicted in Figure 1.

The dataset is essential for analyzing the proposed methodology, so it is gathered from the open-source system. The Mini–Mammographic Image Analysis Society (Mini–MIAS) dataset [22], the Digital Database for Screening Mammography (DDSM) [23] dataset, and the InBreast dataset [24] are chosen to test the performance of the proposed method.

**Figure 2:**
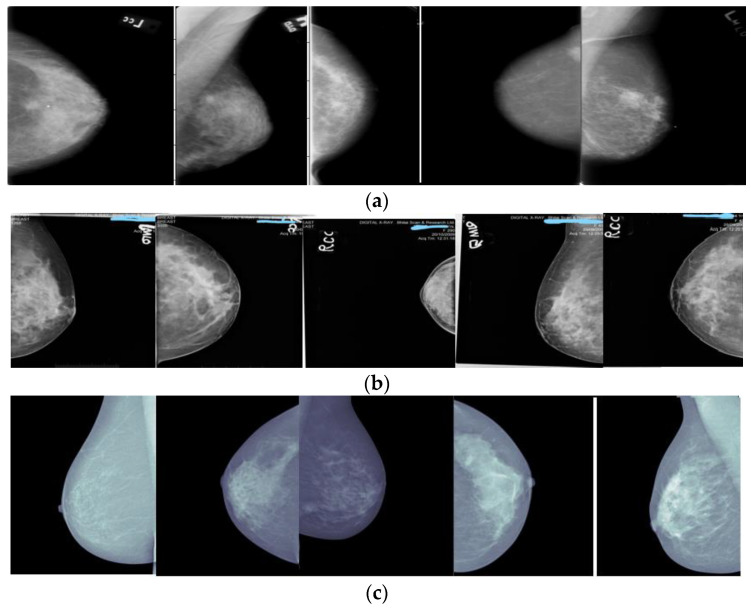
(**a**) DDIS (**b**) MIAS (**c**) InBreast.

Some sample images taken up to show the stages of proposed method is as shown in Figure 2. The pre-processing phase proceeds to eliminate the undesirable noise from the images. The pre-processing image is sent to the detection phase which detects the lumps from the mammographic images. A detailed description of pre-processing stage and detection stage is explained below section.

### 3.1. Stages of Pre-Processing

**Figure 3:**
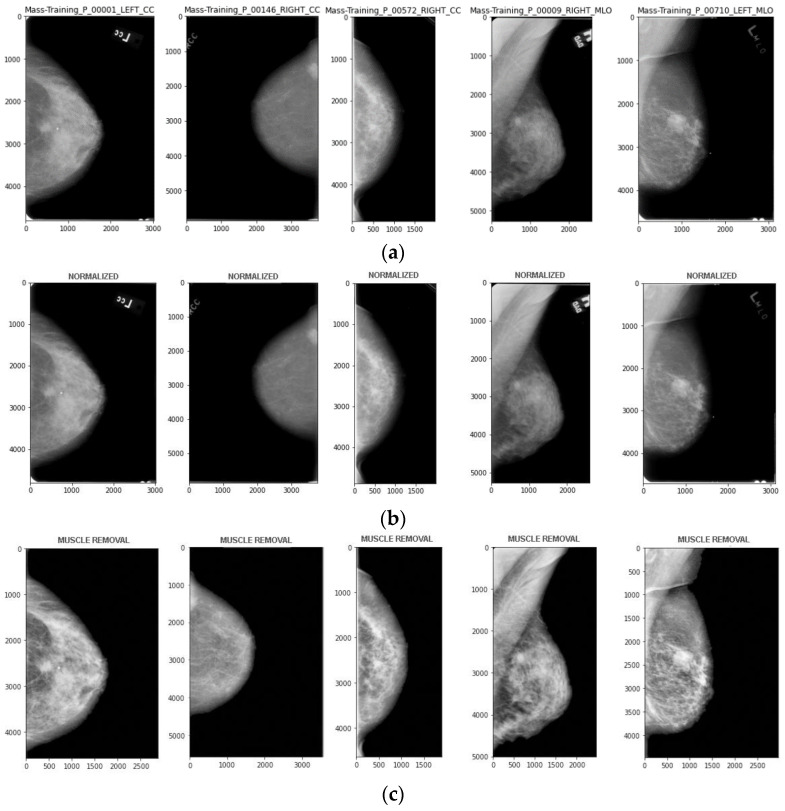
Image pre-processing stages. (**a**) Original image; (**b**) Normalized Image; (**c**). After pectoral Muscle Removal.

During the stage of pre-processing, the unwanted information from the MRI image is removed from the images, which improves the quality of the images. The pre-processing methods of the proposed methodology include removing noise like cropping borders and unwanted information, Min-Max normalization is performed to take up intensity normalization; the pectoral muscle is removed from the resulting image. Since images of two varying views, such as the mediolateral oblique (MLO) view and cranial caudal (CC) view, are considered, the preprocessing steps may slightly differ, as removing pectoral muscles does not happen with cranial caudal view images. Figure 3 shows the stages of preprocessing.

(a) Intensity normalization

In the breast cancer MRI image, the main drawback of the images is that similar kinds of tissue do not have an exact concentration. Various MRI arrangements provide various intensity parameters for the similar tissue type level inside a similar object. This kind of intensity change gives difficult operation of image analysis and detection process. Hence, intensity normalization is the required portion of MRI analysis [25]. Here, Gaussian intensity normalization is utilized, which changes the intensity parameters through a worldwide linear clambering process. This technique is working by main concentrations alienated through the standard deviation of the entire intensity parameters inside which is formulated as follows:

(1) Inew=1σ

where *I* can be described as a primary intensity value, and *σ* can be described as the standard deviation of an entire scan. Based on this process, the detection of the MRI image can be achieved in the range of [0,1024], without any important information loss.

(b) Pectoral Muscle removal

Flipping the image depends on the angle of scan image. CC/MLO decides if the image flip is required. Since breast images are always intended to be in the lower left quadrant of the frame, pixels in the top half are compared with that of the bottom half to determine if the flip is actually needed. For CC images, flipping is conducted if the sum of the top half of image is greater than that of the bottom half. For MLO images, the location of pectoral muscles are first determined since the location of pectoral muscle can change if the orientation of image changes. If the pectoral muscle is located in the top left or right corners, then the image is right side up; otherwise if the pectoral muscle is located in the bottom left or right corners, then the image needs to be flipped vertically.

### 3.2. Intuitionistic Fuzzy C-Means Clustering of Type2

The clustering method [26] is utilized to detect breast cancer disease from MRI images. The projected clustering technique is an advancement of Fuzzy C means Clustering (FCM) and handles more uncertainty in data than FCM [27,28]. In the proposed methodology, two different fuzzifier are utilized which are defined as a fuzzy degree. This proposed fuzzy degree is mathematically formulated as follows:

(2) $$J^{1}\left(U,V\right)=\sum_{I=1}^{N}\sum_{J=1}^{C}U_{IJ}^{M^{2}}\left(X^{I}\right)D^{2}\left(X^{I},V^{J}\right)$$

where $D^{2}\left(X^{I},V^{J}\right)$ can be described as Euclidean distance among the *j*th cluster center and *i*th pattern; $U_{IJ}^{M^{2}}\left(X^{I}\right)$ can be described as the membership function of the *i*th pattern related to the *j*th cluster; *C* can be described as several clusters; and *N* can be represented by several data $X=\left\{X^{1},X^{2},\ldots ,X^{N}\right\}$. In the proposed clustering, the lower and upper membership functions are mathematically formulated as follows:

(3) UIJ→(XI)=max∑K=1CDXI,VJDXI,VK2M1−1−1,   ∑K=1CDXI,VJDXI,VK2M2−2−1

To validate the minimum value in addition to the maximum value of the *j*th cluster center, the Karnik mendal iterative algorithm is utilized. While the iterative algorithm is performed, the right memberships and left memberships are calculated related to each feature for a pattern which is formulated as follows:

(4) $${\left(U^{IJ}\right)}^{L}(X^{I})=\frac{\sum_{L=1}^{M}U^{IL}}{M},\;U^{IL}=\left\{\begin{array}{l} \vec{U^{IJ}}\left(X^{I}\right),\;\;\;if\;X^{IL}\;uses\;\vec{U^{IJ}}\left(X^{I}\right)\;for\;\;V_{J}^{L}\;\\ \underline{U^{IJ}}\left(X^{I}\right),\;\;\;\;\;\;\;\;\;\;\;\;\;\;\;\;Otherwise \end{array}\right.$$

(5) $${\left(U^{IJ}\right)}^{R}(X^{I})=\frac{\sum_{L=1}^{M}U^{IL}}{M},\;U^{IL}=\left\{\begin{array}{l} \vec{U^{IJ}}\left(X^{I}\right),\;\;\;if\;X^{IL}\;uses\;\vec{U^{IJ}}\left(X^{I}\right)\;for\;\;V_{J}^{R}\;\\ \underline{U^{IJ}}\left(X^{I}\right),\;\;\;\;\;\;\;\;\;\;\;\;\;\;\;\;Otherwise \end{array}\right.$$

where *M* can be described as the number of features of a pattern; $V_{J}^{L}$ can be described as a minimum value of *j*th cluster center; $V_{J}^{R}$ can be described as a maximum value of *j*th cluster center; ${\left(U^{IJ}\right)}^{R}$ can be described as right membership functions; and ${\left(U^{IJ}\right)}^{L}(X^{I})$ can be described as left membership function. The membership matrix and crisp centroids can be achieved with the consideration of defuzzification methods and type reduction methods.

(6) $$V^{J}=\frac{V_{J}^{L}+V_{J}^{R}}{2}$$

(7) $$U^{IJ}(X^{I})=\frac{{\left(U^{IJ}\right)}^{L}\left(X^{I}\right)+{\left(U^{IJ}\right)}^{R}\left(X^{I}\right)}{2}$$

With the help of proposed clustering methods, breast cancer images are detected from the MRI images. The cluster center is an essential parameter for achieving the best cluster of the detection process. The optimal cluster center is selected based on the COA progression. The suggested COA-based cluster center selection is described in full in the section below.

### 3.3. Chimp Optimization Algorithm

The COA is used in this proposed technique to choose the best cluster center. The mathematical formulation of the COA is presented as follows:

- Inspiration

Usually, the separating collective community is the common people of a sim. It is considered one of the social orders; the composition of the community may be the work of time variation. Furthermore, in the eyes of the public, each area has a distinct mission and capacity that may alter over time. From this point of view, the point of autonomous ideas is formed in this calculation. As a result, each flock of chimpanzees attempts independently to observe the hunting ground with its specific characteristics planned with a focus on open duty. On a larger scale, four types of chips are introduced: attacker, chaser, border, and driver. Considering these types, the practices of the chips in the hunting system are changed for the production chase action. The drivers collected the prey without resorting to hunting, according to the chimp’s estimations. Boundaries form themselves in vegetation to divert a dam into a prey leak path [29]. Prey is quickly obtained by chasers. Finally, the invaders recognize the departure trend of the prey below the second-rate overlap. Invaders need to be more effective in differentiating the process change of prey. In addition, following a successful chase, the invaders gathered a considerable amount of meat. The attack strategy is fully identified with actual ability, intellect, and age in the chimp assessment. Similarly, monkeys can switch duties during a series or collaborate on their entire procedure. The installation of meat in exchange for social distinctions like planning and concrete help has been accepted by chimp chase. Thus, chimps enjoy the benefit of comparison with other social predators.

- Track and dynamic the prey

Prey can be chased in the COA through exploitation and exploration stages. Furthermore, the numerical scheme of driving the prey is described as follows:

(8) $$D=\left|c\cdot x^{prey}(T)-M\cdot x^{chimp}(T)\right|$$

(9) $$x^{chimp}(T+1)=x^{prey}(T)-A\cdot D$$

where, $x^{prey}$ and $x^{chimp}$ can be represented as the position vector of the chimp and prey, while T can be represented as the various current pressures and the coefficient vectors *A*, *M*, and *C*. COA status vectors are recorded under the condition,

(10) $$A=2\cdot F\cdot R^{1}-a$$

(11) $$C=2\cdot R^{2}$$

(12) M=Choatic  value

where $R^{1}$ and $R^{2}$ can be represented as irregular boundaries, which, in the [0,1] classification, can be represented as the F coefficient, which is directly reduced by 2.5 (0) in the iteration method (in both trials such as exploitation and exploration). M can be represented as a chaotic boundary formed by various chaotic guides. Then, the vector depicts the sexual stimulus behavior of the chimps in the shooting behavior. In the following section, the general representation of the vector value is clarified.

- Investigation phase

The attack behavior of the numerical model of the chimp is planned as follows: to the right of the bat, the chimps can give the area of prey; besides, they can circle it. For a long time, predators were often maintained by invaders. In most chases, the chaser, border, and driver are all involved. There was no information about the prey’s ideal condition during the initial inspection at the examination stage. The attacker’s location should be used to update the chaser, square, and drive positions. As a result, the four best arrangements may be saved, and the various chimps can be stopped to update the levels associated with the best chimps’ components. The following is a numerical introduction to this work:

(13) $$d^{Attac\ker}=\left|C^{1}X^{attac\ker}-M^{1}D\right|$$

(14) $$d^{Barrier}=\left|C^{1}X^{barrier}-M^{2}X\right|$$

(15) $$d^{Chaserr}=\left|C^{3}X^{Chaser}-M^{3}X\right|$$

(16) $$d^{Driver}=\left|C^{4}X^{Driver}-M^{4}X\right|$$

(17) $$X^{1}=X^{Attac\ker}-A^{1}\left(d^{Attac\ker}\right)$$

(18) $$X^{2}=X^{Barreir}-A^{2}\left(d^{Barrier}\right)$$

(19) $$X^{3}=X^{Chaser}-A^{3}\left(d^{Chaser}\right)$$

(20) $$X^{4}=X^{Driver}-A^{4}\left(d^{Driver}\right)$$

(21) $$X\left(T+1\right)=\frac{X^{1}+X^{2}+X^{3}+X^{4}}{4}$$

The search agent position is updated at the trial site depending on one more location of a chimpanzee. As a result, the chimp’s final level, which is portrayed as drivers, chasers, obstacles, and attacker levels, is optionally positioned in a circle.

- Development phase

Before the specified time, the chimps will chase the victim through the attack progression even leaving the prey successively away. The value of *f* is specifically defined in the chimp assault system. A vector is similarly decreased in the vector way f and, furthermore, is a self-determined variable at intervals of $\left[-2F,\;\;2F\right]$. Furthermore, COA tracking, restraining, and driving components have enhanced investigative capability, and this may in any case be at risk of minimal catching conditions in the neighborhood. As a result, an investigation is an essential part of achieving better results. The chimps in COA detour to attack the prey and then join forces to attack the prey. Vector is positioned in the numerical scheme of this trademark, hence the different boundaries. To avoid the optimal problem of the neighborhood, the chimps were required to move away starting the prey, which is |a| > 1. The chimps are driven to blend into the prey |a| < 1 region to reach universal optima.

- A phase of exploitation with the help of a social incentive

In COA, chimps’ social motivation and community were linked to meat hunting. The chimp can limit its hunting interactions in the final stage of the chimp hunting process. As a result, they are frantically trying to obtain prey meat for social forces. These attributes of the chaotic map are programmed with the chaotic maps created as follows:

(22) $$X^{chimp}\left(T+1\right)=\left\{\begin{array}{l} x^{prey}(t)-A\cdot D,\;\;\;\;\;\;if\;\;\;\;\mu <0.5\\ Chaotic\;value,\;\;\;\;\;\;\;if\;\;\;\;\;\mu >0.5 \end{array}\right.$$

where μ is represented as an optional number over time. First, they form spontaneous populations of chimps. Besides, all the chimps are optionally separated by various assemblies such as driver, chaser, and obstacle. From that point forward, each chimp level updates the *f* coefficients with the thought of its collection technique. The best prey area differs in the importance of the driver, chaser, range, and attacker. Later, at that time, the levels are updated, separating from the prey, and there is better tuning of *m* and *c*. Additionally, the value of *f* can be changed from 2.5 to 0, which activates the false trend. In the long run, the level of individuality and emphasis is checked, which gives the best results to deal with the consequences.

## 4. Results and Discussion

This section evaluates and supports the proposed technique’s presentation. In this division, proposed process exhibitions are approved through implementation and comparison testing. To recognize the presence of a projected MRI image detection, the proposed strategy is implemented on an Intel Core i5-2450 M CPU 2.50 GHz PC and 6 GB RAM. This strategy is implemented in Python programming. To approve the interpretation of the proposed strategy, data are collected from open-source system. The proposed technique is implemented and approved using performance metrics such as DSC, JSI, Accuracy, specificity, and sensitivity. The proposed cluster technique is used to distinguish breast cancer disease from MRI images.

The confusion matrix is processed according to the limitations that come with it.

- The malignant regions are introduced and shown as detected which is termed as False Negative (FN).
- An actual malignant region is not detected, which is called a False Positive (FP).
- An actual malignant region is not detected, which is called an undiagnosed true negative (TN).
- An actual malignant region is detected as occurred and called as True Positive (TP).

Given the improvement of the confusion matrix rules, the proposed process implementation is evaluated by measurements, which are defined as:

Accuracy: It is classified into precision detected information examples at all times. The equation of accuracy is introduced as follows:

(23) Accuracy=TN+TPTN+FP+TP+FN

Sensitivity: It can be categorized as a ratio of precisely detected positive examples, including positive events of the following:

(24) Recall=TP(TP+FN)

Specificity: It tends to be characterized as the proportion of accurately sectioned negative occasions to add up to negative examples which are formed as follows:

(25) $$Specificity=\frac{TN}{\left(TN+FP\right)}$$

DSC: It is defined as a similarity index computed based on the below equation:

(26) $$DSC=2\frac{\left|S\cap G\right|}{\left|S+G\right|}$$

(27) $$JSI=2\frac{\left|S\cap G\right|}{\left|S\cup G\right|}$$

**Figure 4:**
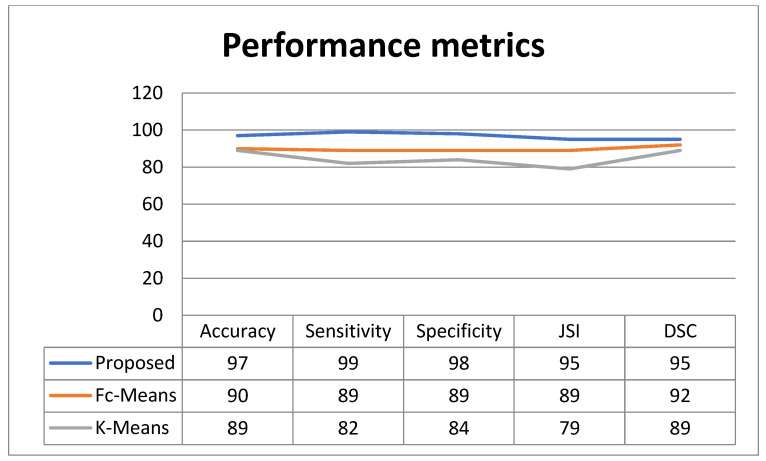
Performance metrics.

The proposed methodology is validated by similarity measurements of DSC and JSI, respectively. Figure 4 depicts the DSC of the proposed technique. From Figure 4, the proposed technique achieved the 0.95% of similarity value of DSC. The conventional methods of F-C mean and K mean clustering achieved 92 in addition to 89. From the comparison analysis, the projected methodology has attained a high similarity value.

From Figure 4, the projected technique achieved the 95% of similarity value of JSI. The conventional methods of F-C mean and K mean clustering achieved 89 and 79. Based on the analysis, the proposed methodology has achieved a high similarity value. The proposed method’s sensitivity is depicted in Figure 4. From Figure 4, the projected technique achieved the 99% of sensitivity. The conventional methods of F-C mean and K mean clustering achieved 89 and 82. Based on the analysis, the projected methodology has attained high sensitivity. The specificity of the proposed methodology is presented in the figure. From the figure, the proposed methodology achieved 98% of specificity. The conventional methods of F-C mean and K mean clustering achieved 89 and 84. Based on the analysis, the proposed methodology has attained high specificity. The accuracy of the proposed methodology is presented in Figure 4. From Figure 4, the proposed methodology achieved 97% of accuracy. The conventional methods of F-C mean and K mean clustering achieved 90 and 89. Based on the analysis, the proposed methodology has attained high accuracy.

## 5. Conclusions

**Figure 5:**
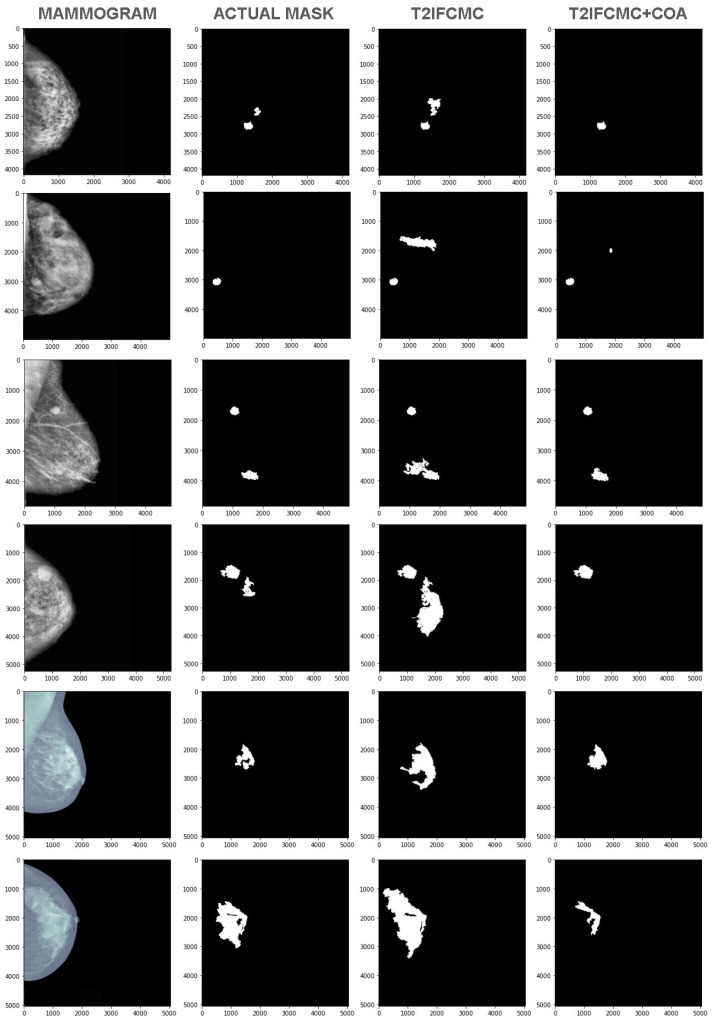
Chimp optimized type 2 intutionistic fuzzy C-means clustering.

In recent years, breast cancer detection is an important task to empower disease prediction as well as classification. In this paper, COA-T2FCM has been designed for detection. The suggested detection method employs a mix of type-2 intuitionistic fuzzy c-means clustering and an oppositional function. The efficient cluster center was selected with the use of the chimp optimization algorithm in type-2 intuitionistic fuzzy c-means clustering. Initially, the type-2 intuitionistic fuzzy c-means clustering objective function has been considered with the consideration of intuitionistic fuzzy information collected from the MRI images. After that, a chimp optimization algorithm is utilized to optimize the cluster center and fuzzifier from the clustering method. The projected technique is implemented in Python as shown in Figure 5, and performances are assessed, such as DSC, JSI, accuracy, sensitivity, and specificity. The proposed method is contrasted with the conventional techniques like fuzzy c means clustering and k mean clustering methods. Based on the investigation, the projected technique has provided the most excellent outcomes parameters of accuracy, specificity, sensitivity, JSI, and DSC, respectively. In the future, the novel proposed technique will be designed to empower the segmentation accuracy of medical images.

## Figures and Tables

**Table 1:** Comparison of various Machine learning/Deep learning-based methods for breast cancer image segmentation with the database taken up and their resulted accuracy.

Reference	ML/DL Methods	Database	Accuracy
[17]	Random forest, NB, SVM, and KNN	MIAS and DDSM	96.47%
[18]	Fast Forward Neural Network	MIAS	93.90%
[19]	SVM using (RBFK)	mini-MIAS	96.92%
[20]	Multilayer perceptron neural network	DDSM	95.60%
[21]	ELM and SVM	mini-MIAS + DDSM	96.02%

## Data Availability

https://www.kaggle.com/datasets/kmader/mias-mammography (accessed on 15 August 2022). http://www.eng.usf.edu/cvprg/mammography/database.html (accessed on 15 August 2022). https://www.kaggle.com/datasets/martholi/inbreast(accessed on 15 August 2022).
